# Delayed refixation of a lesser tuberosity avulsion fracture in a young male with open growth plate: successful repair four years after missed injury

**DOI:** 10.1186/s12891-026-09955-y

**Published:** 2026-05-13

**Authors:** Erdem Koç, Cagman Seker, Alahan Alkis Sensoy, Doruk Akgün, Alp Paksoy

**Affiliations:** 1 Department of Orthopedics and Traumatology, Bilecik Training and Research Hospital, Bilecik, Turkey; 2 Center for Musculoskeletal Surgery, Charité-Universitätsmedizin Berlin, Corporate Member of Freie Universität Berlin and Humboldt-Universität zu Berlin, Augustenburger Pl. 1, Berlin, 13353 Germany

**Keywords:** Open growth plate lesser tuberosity, Fracture, Rotator cuff, Adolescent trauma, Open growth plate

## Abstract

**Background:**

Isolated avulsion fractures of the humeral lesser tuberosity in adolescents with open growth plates are rare and frequently overlooked due to subtle clinical and radiographic findings. Delayed diagnosis may result in persistent internal rotation weakness and functional impairment. This report aims to highlight the diagnostic challenges and clinical course of a markedly delayed presentation of this uncommon injury.

**Case presentation:**

A 19-year-old male presented with persistent weakness in shoulder internal rotation. He reported a traumatic shoulder injury at the age of 15 that had not been further evaluated. Clinical examination and imaging confirmed a chronic avulsion fracture of the humeral lesser tuberosity. Surgical treatment was performed using suture anchors in a double-row fixation technique. Postoperative rehabilitation followed a standardized protocol, and functional recovery was assessed clinically over a six-month follow-up period.

By six months postoperatively, the patient had regained full range of motion and restored internal rotation strength. He returned to unrestricted daily activities without pain or functional limitation. No complications were observed during follow-up.

**Conclusions:**

Avulsion fractures of the humeral lesser tuberosity in skeletally immature patients can remain undiagnosed for years due to subtle presentation. Persistent internal rotation weakness after trauma in adolescents should raise suspicion for this injury. Even in cases of markedly delayed diagnosis, appropriate surgical management can result in excellent functional recovery.

**Supplementary Information:**

The online version contains supplementary material available at 10.1186/s12891-026-09955-y.

## Introduction

Avulsion fractures of the humeral lesser tuberosity are uncommon injuries in skeletally immature patients, and the existing literature contains only a small number of documented cases [1, 2]. As described in case reports and reviews, their incidence is very low, with many cases initially being missed [3]. According to the literature, more than half of the reported cases have been presented as chronic injuries occurring six months after the initial trauma [4], and the reported intervals for diagnosis range from one week to six years (mean 45 weeks; median 12 weeks) [5]. This rarity and the frequent delays in diagnosis underscore the need for a high index of suspicion in young patients with open growth plates presenting with anterior shoulder pain following trauma, particularly when subscapularis dysfunction is suspected.

The most common injury mechanisms for avulsion of the lesser tuberosity are a forceful internal-rotation contraction or excessive loading of the subscapularis muscle, and forceful external rotation of the abducted arm. Both can cause avulsion of the lesser tuberosity because the not-yet-closed growth plate represents the weakest point [3]. Due to the rarity of this fracture type and its limited visibility on conventional shoulder imaging, the injury is difficult to diagnose, which may result in misdiagnosis or delayed diagnosis and treatment [2]. If there is suspicion of abnormality in the lift or abdominal pressure test, anterior-posterior (AP), lateral, and axillary radiographs may not be sufficient for the diagnosis of this fracture. In chronic presentations, as in the present case, magnetic resonance imaging (MRI) and/or computed tomography (CT) should be taken [6–8]. Especially in chronic cases, the avulsed bone fragment can be mistaken as an osteochondroma or neoplasm [2, 9].

In the present case report, an initially undiagnosed lesser tuberosity fracture presenting four years after the initial trauma in a 19-year-old patient is described.

## Case report

### Preoperative assessment

A 19-year-old male patient admitted to the outpatient clinic with internal rotation weakness of the right shoulder. The patient reported having fallen four years earlier while skateboarding, landing on the right shoulder in an abducted position. Initially no further medical consultation was sought. The pain and discomfort resolved after few weeks and the patient continued to do his daily activities and sports. Although internal rotation strength was reduced, the patient assumed it would improve spontaneously. After four years of persistent internal-rotation weakness, he sought evaluation by a shoulder specialist; an external diagnostic workup had raised suspicion of chondromatosis, and he was referred with a preliminary diagnosis of a neoplasm.

**Fig. 1:**
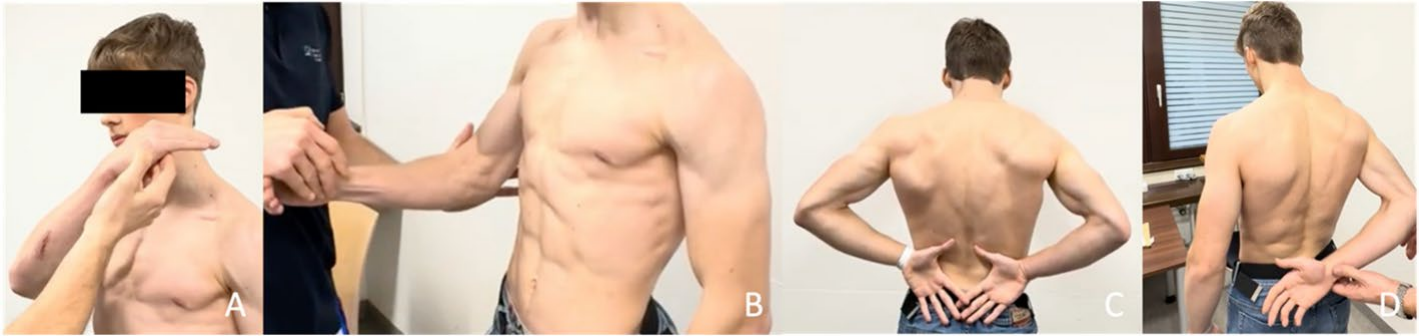
19-year-old male patient with lesser tuberosity avulsion fracture, positive bear-hug test **A**, positive belly press test **B**, preserved internal rotation range of motion (**C**) and positive internal rotation lag test (**D**)

On physical examination, the internal rotation strength was significantly reduced compared to the contralateral side with positive bearhug test, belly press test, and internal rotation lag sign (Fig. 1). There were no range of motion limitations and no loss of strength in abduction, flexion and external rotation.

**Fig. 2:**
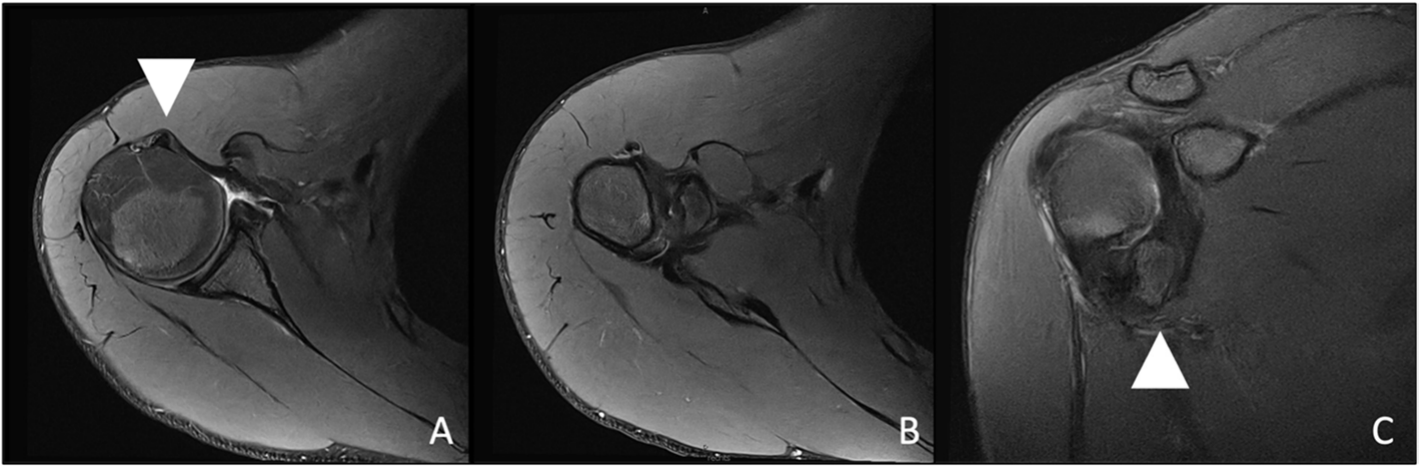
Axillary (**A**-**B**) and coronal (**C**) MRI views of a 19-year-old male with a lesser tuberosity avulsion fracture, subluxated biceps tendon (**A**), axillary nerve (**C**) highlighted by the triangular marker

**Fig. 3:**
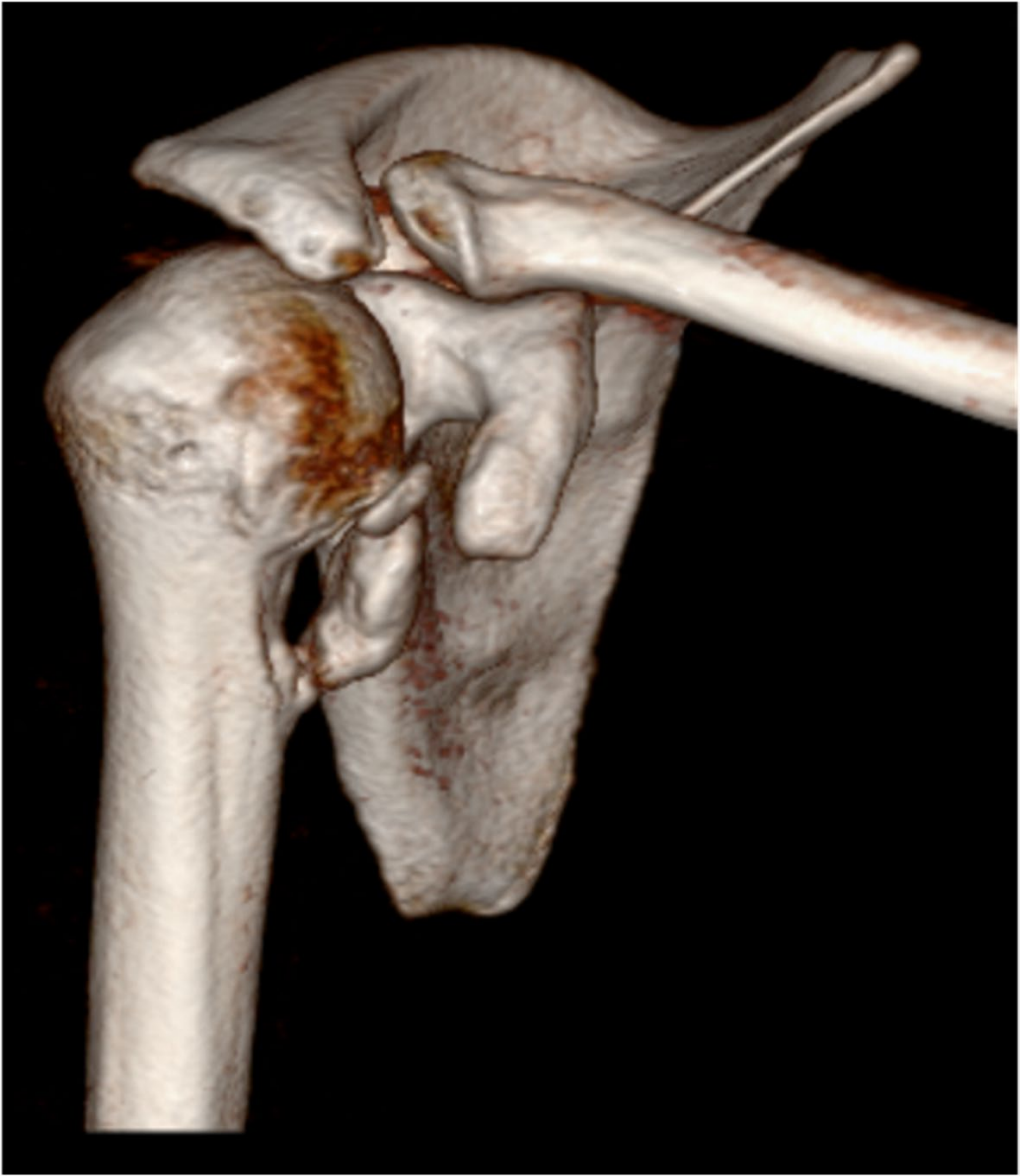
3D reconstruction of the CT-scan

MRI demonstrated medial and distal displacement of the avulsed lesser tuberosity to the level of the pectoralis major insertion. The bony fragment showed soft tissue attachments to the humeral diaphysis as well as to the pectoralis major muscle tendon. Furthermore, the long head of the biceps was luxated from the groove and the axillary nerve traveled only 1–2 cm underneath the inferior tip of the avulsed fragment (Fig. 2). CT imaging demonstrated that the avulsed lesser tuberosity had no bony ingrowth with the humeral diaphysis (Video 1, Fig. 3).

### Surgical procedure

After detailed evaluation of the patient the decision was made to mobilize the avulsed lesser tuberosity fragment and perform a refixation with suture anchors in double-row technique.

**Fig. 4:**
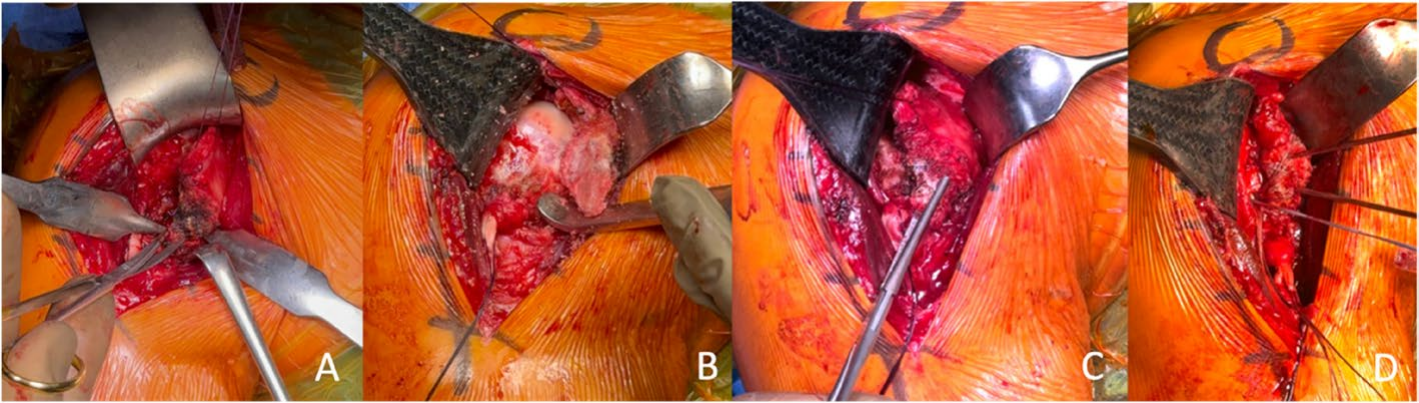
Peropative photographs of the surgery, dissection of avulsed lesser tuberosity fragment (**A**), mobilization of fragment (**B**) and subscapularis muscle (**C**), fixation of lesser tuberosity with anchors (**D**)

Surgery was performed under general anesthesia and in beach chair position. The deltopectoral approach was used. After retraction of the conjoined tendon, the subscapularis muscle and the avulsed lesser tuberosity was identified. Then, a careful dissection of the avulsed fragment from the humerus, the pectoralis major tendon, and the adjacent soft tissues was performed. Care was taken to avoid damage the axillary nerve, which was just few centimeters inferior from the inferior tip of the fragment. Then the long head of the biceps was tenotomized from the glenoid insertion site and a soft tissue tenodesis was performed. The avulsion site of the proximal humerus as well as the avulsion surface of the lesser tuberosity were debrided with the help of a burr. First, two suture anchors (BioComposite Corkscrew FT Anchor 5.5 mm, loaded with No. 2 FiberWire; Arthrex, Naples, Florida, USA) were placed medially to the bone cartilage border and all the sutures are passed through the subscapularis tendon in mattress fashion. Then a temporary reduction of the lesser tuberosity was performed and temporarily fixed with two 2.0 mm K-wires. After reduction assessment via fluoroscopic imaging the medial row was knotted. While keeping the K-wires in place the sutures from the medial row were then anchored laterally to the bicipital groove using two 4.75 mm SwiveLock anchors (Arthrex, Naples, Florida, USA) in terms of a double-row reconstruction (Fig. 4, Video 2). The K-wires are removed, and the wound is closed in a typical fashion.

### Postoperative rehabilitation

Postoperatively, an arm sling in internal rotation was applied for six weeks allowing passive exercises with abduction and external rotation restrictions. After six weeks the sling was discontinued and after radiologic assessment active range of motion was started.

### Follow-up evaluations

**Fig. 5:**
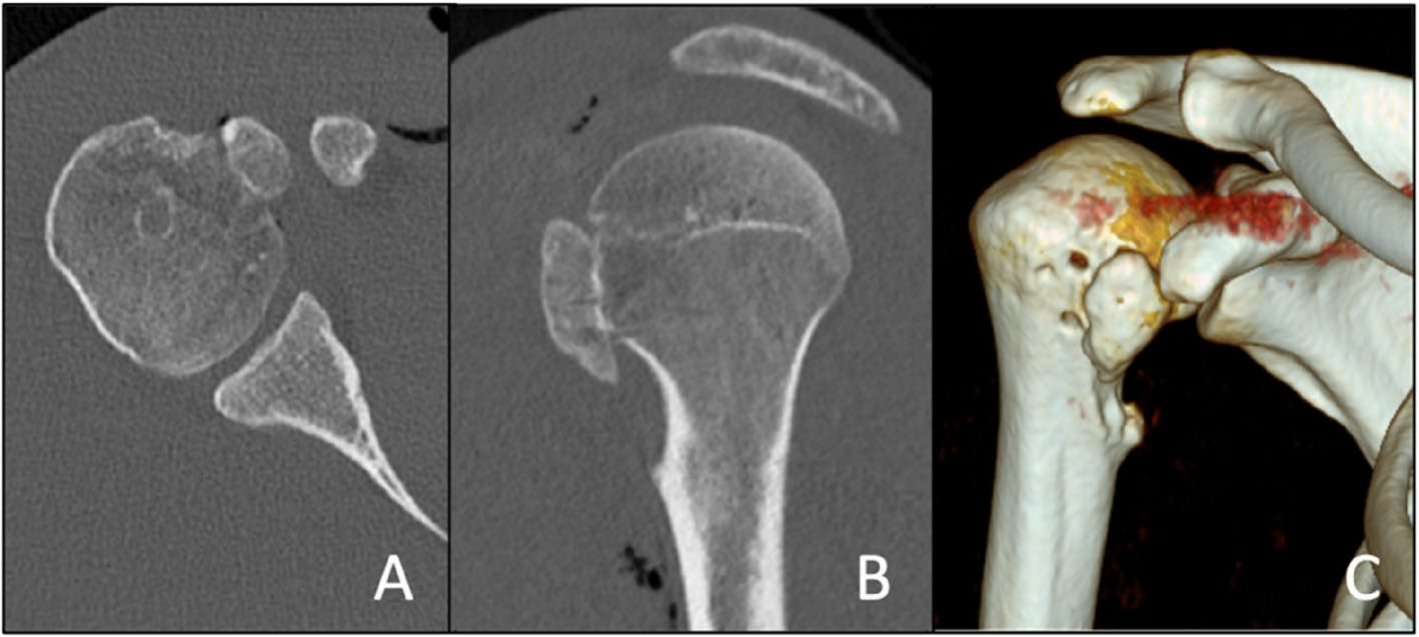
Postoperative axial computed tomography (CT) image in axial (**A**) and sagittal views (**B**), and 3D CT image (**C**)

**Fig. 6:**
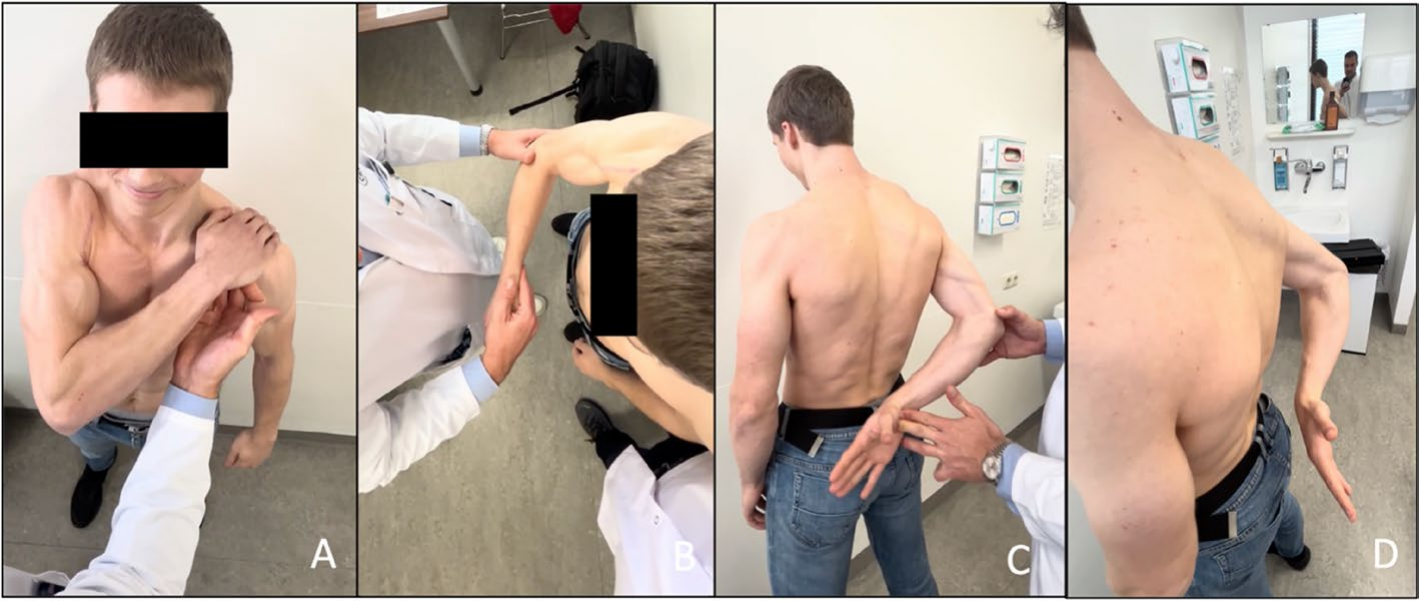
Postoperative sixth month follow-up, negative bearhug test (**A**), negative belly press test (**B**), negative lift off test (**C**) and negative internal rotation lag sign (**D**)

At six months postoperatively, the patient was able to perform all daily activities without limitation. Examination showed that internal rotation was comparable to the healthy contralateral side in terms of range of motion and strength, and all signs of internal-rotation deficit were negative. The radiological imaging and physical examinations during follow-up showed a bony consolidation of the lesser tuberosity and are illustrated in Figs. 5 and 6. The patient’s American Shoulder and Elbow Surgeons score was 93 and Constant-Murley score was 94 at the sixth month postoperative follow up. Written informed consent was obtained from the patient for publication of this case and images.

## Discussion

This case demonstrates that lesser tuberosity avulsion fractures in young patients can remain undiagnosed for extended periods and yet achieve optimal functional outcomes following surgical intervention. Despite the occurrence of a four-year delay, fragment displacement, and the presence of extensive soft tissue adhesions, the implementation of double-row suture anchor refixation resulted in the restoration of nearly complete internal rotation strength. It is imperative to note that this case underscores the notion that chronicity alone should not preclude operative intervention, and that advanced imaging is paramount when subscapularis dysfunction is suspected in young patients presenting with anterior shoulder pain following trauma.

The proximal humerus develops from three ossification centers—the articular surface, greater tuberosity, and lesser tuberosity—which merge in early childhood (5–7 years) and fuse with the humeral shaft during adolescence (14–17 years in girls, 16–18 years in boys), with full maturation by late teenage years [10–12]. The lesser tuberosity is the last region to complete ossification [13]. In skeletally immature patients, the physis and apophysis form the weakest part of the tendon–bone interface, making the lesser tuberosity prone to traction-related avulsion [7].

In avulsion fractures of the lesser tuberosity, the prevailing mechanism involves a forceful contraction of the subscapularis tendon during shoulder abduction and external rotation, which can lead to avulsion of the apophysis in adolescents [14]. Some reports highlight non-traumatic triggers such as electrocution, further emphasizing that muscular force rather than direct impact is responsible in many cases [4]. Radiographically, diagnosis can be challenging as standard X-rays may miss even displaced fragments. CT or MRI scan are often necessary to reliably detect the lesion [14]. According to the literature delayed diagnosis is indeed common and may worsen outcomes [14–16].

The extant literature indicates that treatment options for such cases include conservative management, open reduction and internal fixation (ORIF), and arthroscopic or suture-anchor fixation. In younger patients in whom the fragment is minimally displaced and there is minimal soft-tissue injury, conservative management has been shown to be an effective treatment [17]. Several authors advocate surgical fixation in displaced fractures to restore subscapularis function and avoid malunion or persistent weakness [4, 14–17]. For instance, Chang et al. documented a case that was treated with ORIF and exhibited full range of motion within a three-month period, and had symptom-free full range of motion in the first postoperative year [1]. In this case, double-row fixation is the preferred due to the fact that it facilitates a more effective restoration of the native subscapularis footprint, in addition to providing enhanced compression and biomechanical stability. This, in turn, may promote enhanced healing in chronic cases where tissue quality has been compromised [18]. 

The prevailing tendency in the existing literature is to report favorable outcomes when the injury is recognized and managed appropriately. In most cases, this results in a return of full range of motion and strength within a period of three to six month [5, 16]. However, delayed diagnosis may result in complications such as nonunion, malunion, persistent subscapularis weakness or loss of external rotation, long-head biceps pathology, and overall shoulder dysfunction [15]. The review by Tosun and Kesemenli underscores the significance of timely surgical intervention in displaced cases for achieving optimal outcomes [16].

A distinctive feature of this case is the remarkable time interval of four years between the initial shoulder injury and the definitive diagnosis of lesser tuberosity avulsion in an adolescent patient with open physis. This represents one of the longest diagnostic delay reported in the literature to date. Previous reports have documented delays ranging from several weeks to a few months, with exceedances of one year being rare (one week to six years; mean 45 weeks; median 12 weeks) [5]. Notably, despite the prolonged delay before diagnosis, the patient achieved excellent functional recovery following refixation with suture anchors in double-row technique. This favorable outcome suggests that, in selected cases, chronic lesions may still allow successful anatomical restoration, particularly when fragment viability and tendon integrity are preserved. However, such cases are exceptionally rare, and most authors emphasize the importance of early diagnosis and fixation to prevent persistent weakness or malunion [16]. Consequently, the present findings serve to expand the known clinical spectrum of this rare injury, thereby illustrating that satisfactory outcomes can still be obtained despite extreme diagnostic delay.

## Conclusion

The incidence of lesser tuberosity fractures in pediatric patients with open growth plates is low, and the diagnosis of such fractures can often be challenging due to the absence of overt clinical signs and the presence of subtle radiographic findings. Maintaining a high index of suspicion and using appropriate imaging in patients with posttraumatic shoulder internal rotation limitation are essential for timely recognition. In patients with avulsed bone fractures, especially those with chronic symptoms, should be considered that this fragment may be confused with a neoplasm. Accurate diagnosis and appropriate treatment can yield excellent functional outcomes, even in cases of delayed presentation.

## Supplementary Information

Supplementary Material 1. Video 1: CT imaging of a 19-year-old male with a lesser tuberosity avulsion fracture, showing the coronal (A), axial (B) and sagittal (C) CT views. Video 2: Double-row refixation of the lesser tuberosity.

## Data Availability

All data generated or analysed during this study are included in this published article.

## Abbreviations

AP: Anterior-posterior
CT: Computed tomography
MRI: Magnetic resonance imaging
ORIF: Open reduction and internal fixation
