# Supplementary material for: Delayed refixation of a lesser tuberosity avulsion fracture in a young male with open growth plate: successful repair four years after missed injury
Source: BMC Musculoskelet Disord. 2026 May 13;27:417. doi: 10.1186/s12891-026-09955-y (PMC13179633; doi:10.1186/s12891-026-09955-y)
Supplement: Supplementary file 1 — Supplementary Material 1. Video 1: CT imaging of a 19-year-old male with a lesser tuberosity avulsion fracture, showing the coronal (A), axial (B) and sagittal (C) CT views. Video 2: Double-row refixation of the lesser tuberosity. [file 12891_2026_9955_MOESM1_ESM.docx]

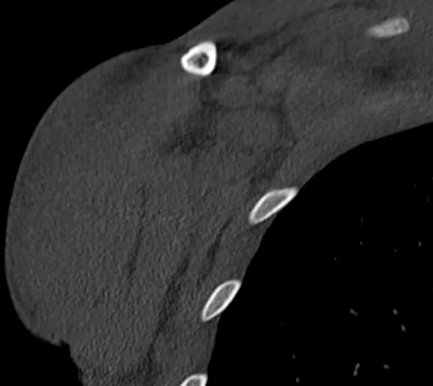

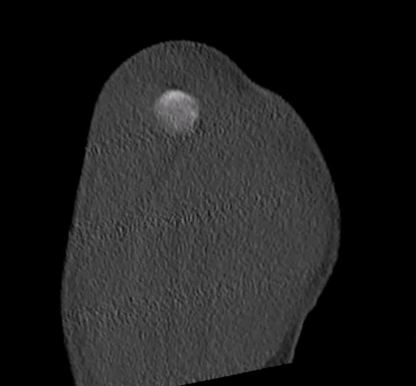

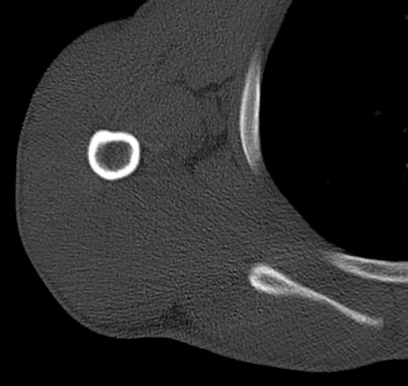
**Video Legends:**


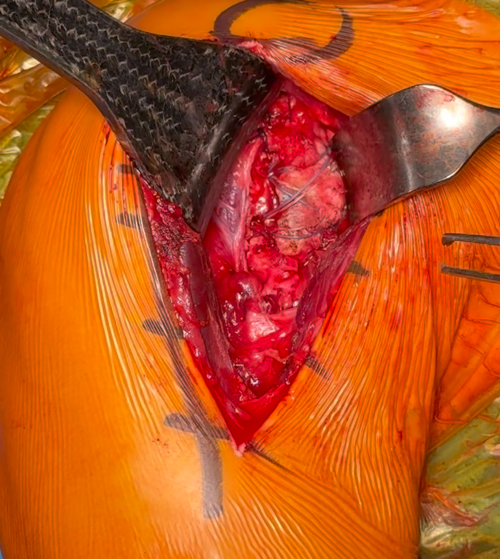
**Video 1** : CT imaging of a 19-year-old male with a lesser tuberosity avulsion fracture, showing the coronal (A), axial (B) and sagittal (C) CT views.

**Video 2:** Double-row refixation of the lesser tuberosity
